# Limited effect of leg elevation in preventing intraoperative hypotension during total shoulder arthroplasty in the beach chair position: a randomized controlled trial

**DOI:** 10.1186/s12871-025-03299-1

**Published:** 2025-10-01

**Authors:** Seungpyo Nam, Seokha Yoo, Sae Hoon Kim, Sun-Kyung Park, Young-Jin Lim, Jin-Tae Kim

**Affiliations:** 1 Department of Anesthesiology and Pain Medicine, Seoul National University Hospital, Seoul National University College of Medicine, Seoul, Republic of Korea; 2 Department of Orthopedic Surgery, Seoul National University Hospital, Seoul National University College of Medicine, Seoul, Republic of Korea; 3 Department of Anesthesiology and Pain Medicine and Anesthesia and Pain Research Institute, Yonsei University College of Medicine, Seoul, Republic of Korea

**Keywords:** Hypotension, Hemodynamic monitoring, Patient positioning, Intraoperative complication, Intraoperative care, Total shoulder replacement

## Abstract

**Background:**

The beach chair position, often used in shoulder surgery, increases the risk of hypotension and cerebral hypoperfusion. This randomized controlled trial evaluated the efficacy of leg elevation in preventing hypotension during total shoulder arthroplasty in the beach chair position.

**Methods:**

Fifty patients scheduled for total shoulder arthroplasty in the beach chair position were randomly assigned to the control (25 patients) or treatment (25 patients) groups. The treatment group elevated their legs during surgery, whereas the control group maintained a neutral leg position. The primary outcome was the incidence of hypotension. The secondary outcomes included the incidence of cerebral desaturation, hemodynamic variables, and vasoconstrictor use.

**Results:**

The incidence of hypotension did not significantly differ between the control and treatment groups (100% [95% CI: 86.3-100%] vs. 87.5% [95% CI: 67.6-97.3%]; *p* = 0.11). The incidence of cerebral desaturation was also similar between the control and treatment groups (84.0% [95% CI: 63.9-95.5%] vs. 91.7% [95% CI: 73.0-99.0%]; *p* = 0.67). However, the median [Q1-Q3] dose of phenylephrine was significantly higher in the control group than in the treatment group (3.5 [1.5–5.4] μg/kg vs. 1.6 [0.9–3.0] μg/kg; *p* = 0.02).

**Conclusions:**

Intraoperative hypotension occurred in 93.9% of patients undergoing total shoulder arthroplasty in the beach chair position, regardless of leg elevation. However, leg elevation significantly reduced the need for vasoconstrictors to maintain blood pressure during anesthesia.

**Trial registration:**

ClinicalTrials.gov (identifier: NCT03393559, date of registration: 2017–12-26).

**Supplementary Information:**

The online version contains supplementary material available at 10.1186/s12871-025-03299-1.

## Introduction

The beach chair position has been widely accepted for shoulder surgery due to its convenience for surgeons, ease of understanding the shoulder anatomy, and its effectiveness in reducing bleeding [1]. However, it carries the risk of hemodynamic instability, including hypotension and decreased cardiac output, due to venous pooling in the lower body [2]. Moreover, the associated gravitational force can further reduce cerebral blood flow, potentially leading to serious complications such as stroke, vision loss, and even brain death [3, 4].

Various preventive strategies have been explored to mitigate the risk of catastrophic complications associated with the beach chair position. A study by Kwak et al. demonstrated that intermittent leg compression was effective in reducing the incidence of hypotension from 64 to 28% during the transition from the supine to beach chair position in shoulder arthroscopy [5]. Other previous studies have shown that the prophylactic administration of vasopressors, including phenylephrine and vasopressin, can attenuate hypotension in this position. However, these strategies were not effective in preventing cerebral desaturation [6, 7]. These findings underscore the need for additional strategies that address intravascular volume status to prevent cerebral hypoperfusion during beach chair positioning.

Leg elevation or passive leg raising is a simple, non-invasive intervention traditionally used to prevent hypotension, particularly during spinal anesthesia [8, 9]. By facilitating venous return from the lower extremities, it can transiently improve cardiac preload and stabilize hemodynamics. However, while this technique has shown benefits in obstetric and neuraxial anesthesia settings, its effectiveness during general anesthesia in the beach chair position remains underexplored.

This randomized controlled trial evaluated whether leg elevation, as a standalone intervention, could maintain hemodynamics stability and cerebral oxygenation in patients undergoing total shoulder arthroplasty in the beach chair position. We hypothesized that leg elevation would reduce the incidence of intraoperative hypotension and cerebral desaturation.

## Methods

### Ethics

This prospective, randomized, controlled study was approved by the Institutional Review Board of Seoul National University Hospital (No. 1712–023-903) on December 22, 2017, and was registered on ClinicalTrials.gov before recruitment of the first participants (NCT03393559). The study adhered to CONSORT guidelines, with a completed CONSORT checklist provided as a supplementary file. It was conducted in compliance with the Declaration of Helsinki, and written informed consent was obtained from all participants before surgery.

### Study population and randomization

The study involved healthy adult patients aged 19 to 80 years who were scheduled for total shoulder arthroplasty in the beach chair position under general anesthesia. Exclusion criteria included patient refusal, coronary artery disease, cerebrovascular disease, autonomic nerve disorder, body mass index (BMI) > 30 kg/m^2^, inability to perform invasive blood pressure monitoring due to conditions such as arrhythmia or pacemaker insertion, and changes in the operation plan. Patients with limitations in hip or knee flexion due to joint disorders were also excluded.

Patients were randomly assigned to two groups based on a computer-generated randomization list: neutral leg position (control group) or elevated leg position (treatment group) during surgery. A randomization list was created using block randomization with a randomly selected block size of two or four in a reproducible sequence. Group allocations were placed in sequentially numbered sealed opaque envelopes and opened after anesthesia induction by a nurse who was not involved in the study. Although patients were blinded to their group allocation, blinding of the investigators was not feasible due to the nature of the intervention; therefore, the study was conducted using a single-blind design.

### Data collection and the study protocol

Routine monitors, including noninvasive blood pressure, 3-lead electrocardiograms, pulse oximeters, and bispectral index monitoring (BIS™, Medtronics, Minneapolis, MN, USA), were applied in the operating room. Following local anesthesia, a 20-gauge arterial catheter was inserted into the radial artery on the opposite side of the surgical field for invasive blood pressure monitoring. The pressure transducer for invasive blood pressure monitoring was positioned at the mid-axillary level when the patients were supine, and at the level of the fifth intercostal space when they were seated. INVOS™ cerebral oximetry sensors (Medtronic, Minneapolis, MN, USA) were placed bilaterally 1–2 cm above the eyebrow according to the manufacturer’s instructions.

Anesesia was induced with an effect-site target-controlled infusion of remifentanil set at 3.0 ng/mL and 1–2 mg/kg of propofol in bolus. During induction, a rapid infusion of approximately 500 mL of isotonic crystalloids such as lactated Ringer’s solution was administered. After confirming loss of consciousness, 0.8 mg/kg of rocuronium was administered to facilitate tracheal tubation. Following successful intubation, the lungs were mechanically ventilated with an oxygen/air mixture (fraction of inspired oxygen, 40%), a tidal volume of 8 mL/kg of ollopredicted body weight, and a respiratory rate of 12 breaths per minute to maintain end-tidal carbon dioxide tension between 35 and 40 mmHg. Anesthesia was maintained using sevoflurane and remifentanil, targeting a BIS of 40–60. Intraoperative maintenance fluids were infused continuously at a rate of 100 mL/hr. In the event of intraoperative bleeding, 3 mL of isotonic crystalloid solution was administered for every 1 mL of blood loss.

**Fig. 1:**
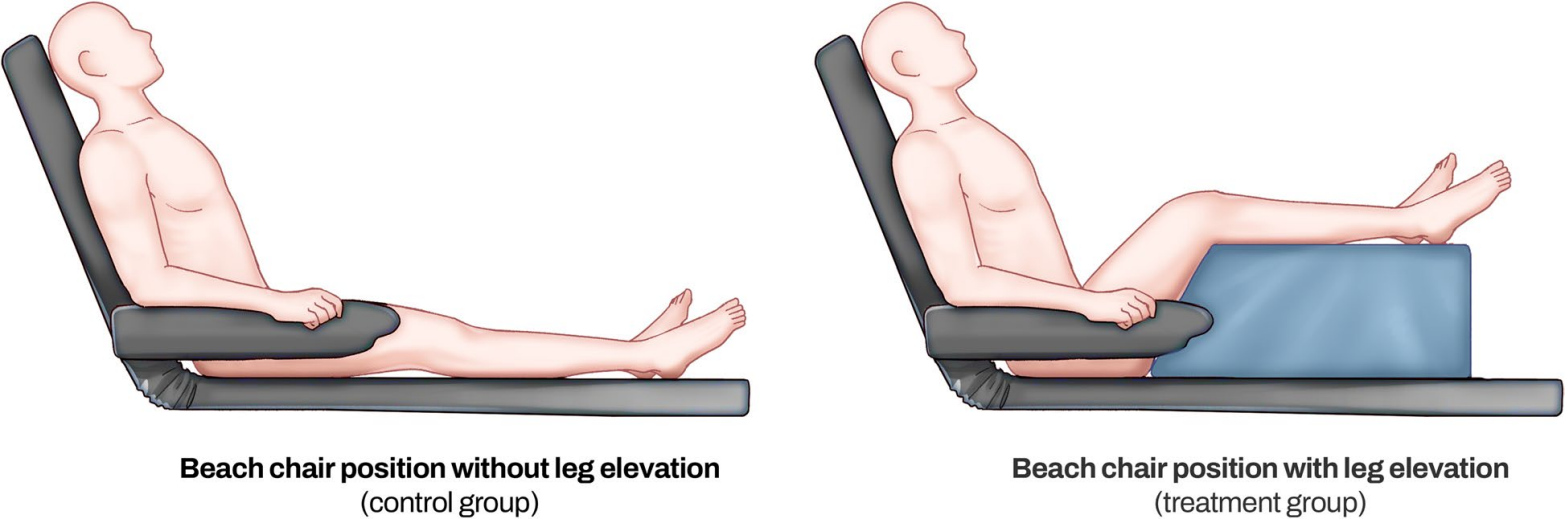
Schematic diagram of the beach chair position

After induction, the patients were positioned in a seated posture using the beach chair module, and their heads were secured with a sponge helmet. Subsequently, the patients assigned to the treatment group were provided with a cushion under their legs to flex their hip and knee joints at a 45-degree angle, positioning the knees at the level of the heart (Fig. 1). In contrast, the control group maintained a seated posture with a soft blanket under their legs to prevent pressure sores during surgery.

All episodes of hypotension and cerebral desaturation that occurred after changing to the beach chair position were recorded and managed by an attending anesthesiologist throughout surgery. Intraoperative hypotension was defined as a mean arterial pressure (MAP) < 60 mmHg or systolic blood pressure (SBP) < 80% of the baseline value measured on the day before surgery for at least one minutes. In the event of intraoperative hypotension, patients were initially administered intravenous phenylephrine at a dose of 0.5 µg/kg. If hypotension persisted after one minute, the dose was increased to 1.0 µg/kg and re-administered. If hypotension continued despite these measures, an additional 10 mg of intravenous ephedrine was administered. In cases of hypotension accompanied by bradycardia (heart rate < 50 beats/min), 10 mg intravenous ephedrine was initially administered, followed by 0.5 µg/kg of phenylephrine if hypotension persisted after one minute. Cerebral desaturation was defined as regional cerebral oxygen saturation (rSO_2_) < 80% of the baseline value for > 30 s. It was managed through the administration of vasopressors, increasing the fraction of inspired oxygen in increments of 10%, and/or blood transfusion, if necessary (hemoglobin level < 8 g/dl).

### Primary and secondary outcomes

Patient demographic data, including previous medical history, long-term medication use, and other comorbidities, were reviewed from electronic medical records. The primary outcome was the incidence of hypotension during the beach chair position. The secondary outcomes included the incidence rate of cerebral desaturation in the beach chair position, total number of hypotensive and cerebral desaturation episodes per patient, hemodynamic variables (systolic blood pressure (SBP), mean arterial blood pressure (MAP), diastolic blood pressure (DBP), heart rate (HR), and regional cerebral oxygen saturation (rSO_2_)) at predetermined time points, and total amount of ephedrine and phenylephrine administered during the surgery. The predetermined time points included: before induction; 1 min before changing to the beach chair position; 1 min, 5 min, 30 min, and 1 h after the position change; 1 min before transitioning to a supine position for anesthesia emergence; and 1 min after changing to the supine position.

### Statistical analyses

The sample size was calculated based on previous studies indicating that the incidence rate of intraoperative hypotension ranges from 53 to 64% among patients undergoing shoulder surgery in the beach chair position [5, 10]. Assuming an incidence rate of approximately 60% in the control group and hypothesizing that leg elevation could reduce this incidence to 20%, it was determined that 22 patients per group were needed to achieve a statistical power of 0.8 and a significance level of 0.05. To account for the potential dropout rate of 10%, 50 patients (25 per group) were included in this study.

Statistical analyses were performed using the R software version 4.2.1 (R Foundation for Statistical Computing, Vienna, Austria). All data are expressed as mean ± SD for normally distributed variables and as median [Q1-Q3] for non-normally distributed variables, unless otherwise specified. Normal distribution was assessed using the Shapiro–Wilk test and Q-Q plot.

Patient characteristics were compared between the groups by calculating the standardized mean difference and 95% confidence interval (CI). Parametric variables, including preoperative SBP, total anesthesia time, total operation time, and total infused fluids, were compared between the groups using independent *t*-tests. Non-parametric variables, including the number of hypotension and cerebral desaturation episodes per patient, total estimated blood loss, and total use of vasopressors (ephedrine and phenylephrine), were compared between the groups using the Mann–Whitney U test. The incidences of intraoperative hypotension and cerebral desaturation were separately analyzed and compared between the groups using Fisher’s exact test.

To investigate whether leg elevation affected hemodynamic variables at time points expected to show fluctuations due to positional changes, a two-way mixed analysis of variance (ANOVA) with repeated measures was performed. The variables analyzed were SBP, MAP, DBP, HR, and rSO_2_. This analysis included time as a within-group factor, group as a between-group factor, and an interaction term between the group and time to determine whether the two groups differed over time. Mauchly’s test was used to assess the assumption of sphericity, and if sphericity was violated, the Greenhouse–Geisser correction was applied. For variables with missing data, a linear mixed-effects model was used for analysis.

Significant main or interaction effects identified in the two-way repeated measures ANOVA were further examined using post-hoc analyses. Within-group comparisons were conducted using paired *t*-tests with Bonferroni correction, whereas between-group comparisons were performed using independent *t*-tests with Bonferroni correction. For the linear mixed effects model, post-hoc comparisons were conducted by calculating estimated marginal means, with appropriate adjustments for multiple comparisons, to discern specific differences between groups or time points. Statistical significance was defined as a two-sided *P* value < 0.05.

## Results

**Fig. 2:**
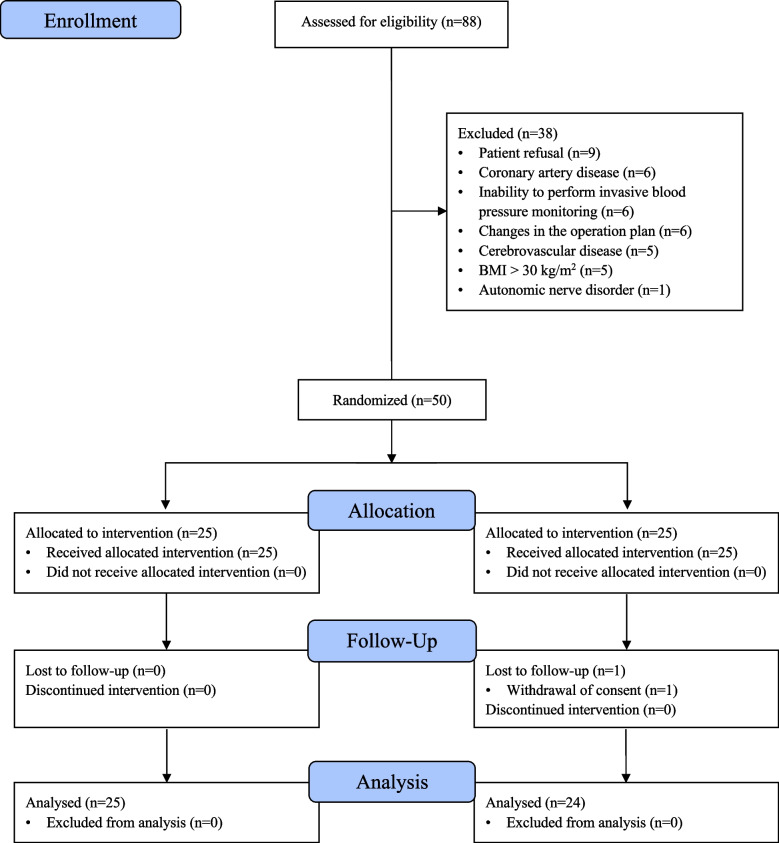
Flow diagram of patient participation

Among the 88 patients screened for eligibility between December 2017 and December 2019, 50 were enrolled, and 49 were analyzed. The control group consisted of 25 patients, whereas the treatment group included 24 patients due to the dropout of one participant (Fig. 2). The patient characteristics are summarized in Table 1. The standardized mean difference and 95% confidence interval for all compared variables between the two groups were calculated to be 0.4 or less, indicating that the differences between the groups were minimal.

**Table 1:** Patient characteristics

	Control group (*n* = 25)	Treatment group (*n* = 24)	SMD (95% CI)
Sex			
Male	5 (20)	4 (16.7)	−0.08 (−0.64, 0.48)
Female	20 (80)	20 (83.3)	0.08 (−0.48, 0.64)
Age (years)	71 [66–79]	74 [68–79]	0.10 (−0.47, 0.66)
Height (cm)	153.4 ± 8.1	154.1 ± 9.2	0.08 (−0.48, 0.64)
Weight (kg)	57.1 ± 10.0	59.3 ± 8.2	0.24 (−0.32, 0.80)
BMI (kg/m^2^)	24.1 ± 3.1	25.0 ± 2.9	0.30 (−0.26, 0.86)
ASA physical status			
I	4 (16)	4 (16.7)	0.02 (−0.54, 0.58)
II	19 (76)	18 (75)	−0.02 (−0.58, 0.54)
III	2 (8)	2 (8.3)	0.01 (−0.55, 0.57)
Underlying diseases			
HTN	15 (60)	12 (50)	−0.20 (−0.76, 0.36)
DM	6 (24)	6 (25)	0.02 (−0.54, 0.58)
Medications			
ARB/ACEi	11 (44)	9 (37.5)	−0.13 (−0.69, 0.43)
CCB	10 (40)	7 (29.2)	−0.22 (−0.79, 0.34)
β blocker	3 (12)	2 (8.3)	−0.12 (−0.68, 0.44)
⍺ 1 blocker	2 (8)	0 (0)	−0.40 (−0.97, 0.16)
Diuretics	2 (8)	0 (0)	−0.40 (−0.97, 0.16)
MFM, OHA	5 (20)	5 (20.8)	0.02 (−0.54, 0.58)

Data are presented as mean ± SD, median [Q1-Q3], number of patients (percentage), or standardized mean difference (95% confidence interval)

*BMI* Body mass index, *ASA* physical status, American Society of Anesthesiologists physical status, *HTN* hypertension, *DM* diabetes mellitus, *ARB/ACEi* angiotensin receptor blocker/angiotensin-converting-enzyme inhibitor; *CCB* calcium channel blocker, *MFM* metformin, *OHA* oral hypoglycemic agent

The primary and secondary outcome variables are shown in Table 2. There was no significant difference in the incidence of hypotension between the control and treatment groups (25/25, 100% [95% CI: 86.3-100%] vs. 21/24, 87.5% [95% CI: 67.6-97.3%]; *p* = 0.11). The incidence of cerebral desaturation also did not differ between the two groups (21/25, 84.0% [95% CI: 63.9-95.5%] vs. 22/24, 91.7% [95% CI: 73.0-99.0%]; *p* = 0.67). Meanwhile, a greater amount of phenylephrine was administered to manage intraoperative hypotension and/or cerebral desaturation in the control group than in the treatment group (3.5 [1.5–5.4] µg/kg vs. 1.6 [0.9–3.0] µg/kg; *p* = 0.02).

**Table 2:** Perioperative measurements and incidences of intraoperative hypotension and cerebral desaturation during the beach chair position for total shoulder surgery

	Control Group (*n* = 25)	Treatment Group (*n* = 24)	*p*
Intraoperative hypotension	25 (100; 86.3–100)	21 (87.5; 67.6–97.3)	0.11
Episodes per patients	5 [3–7]	4 [2–5.5]	0.16
Cerebral desaturation	21 (84; 63.9–95.5)	22 (91.7; 73.0–99.0)	0.67
Episodes per patients	2 [1–3]	2.5 [1–5]	0.20
Preoperative SBP (mmHg)	125 ± 13	126 ± 11	0.71
Total anesthesia time (minutes)	96.2 ± 15.2	97.3 ± 16.7	0.81
Total operation time (minutes)	64.0 ± 15.6	61.7 ± 14.9	0.59
Total infused fluid (ml)	718 ± 301	681 ± 190	0.61
Estimated blood loss (ml)	200 [100–200]	150 [100–300]	0.93
Total dose of ephedrine (mg)	10 [0–20]	0 [0–10]	0.26
Total dose of phenylephrine (µg/kg)	3.5 [1.5–5.4]	1.6 [0.9–3.0]	0.02
Increased FiO_2_	2 (8.0; 1.0–26.0)	1 (4.2; 0.1–21.1)	1.00

Data are presented as mean ± SD, median [Q1-Q3], or number of patients (percentage; 95% confidence interval)

Two-way mixed ANOVA with repeated measures was performed for all hemodynamic variables, except for rSO_2_. The analysis revealed no significant group effects or interaction effects (all *p* > 0.05). However, significant time effects were observed across all hemodynamic variables (all *p* < 0.01), indicating substantial changes in these hemodynamic parameters over time irrespective of the groups. Due to the presence of some missing values in the left and right rSO_2_ measurements, a linear mixed effects model was applied for these variables. The analysis indicated that the group effects and interaction effects between group and time were not significant for either left or right rSO_2_ (both *p* > 0.05). Nevertheless, significant fixed effects of time were observed for both left and right rSO_2_ (all *p* < 0.05), except at T2 (*p* = 0.08 and 0.06, respectively).

**Fig. 3:**
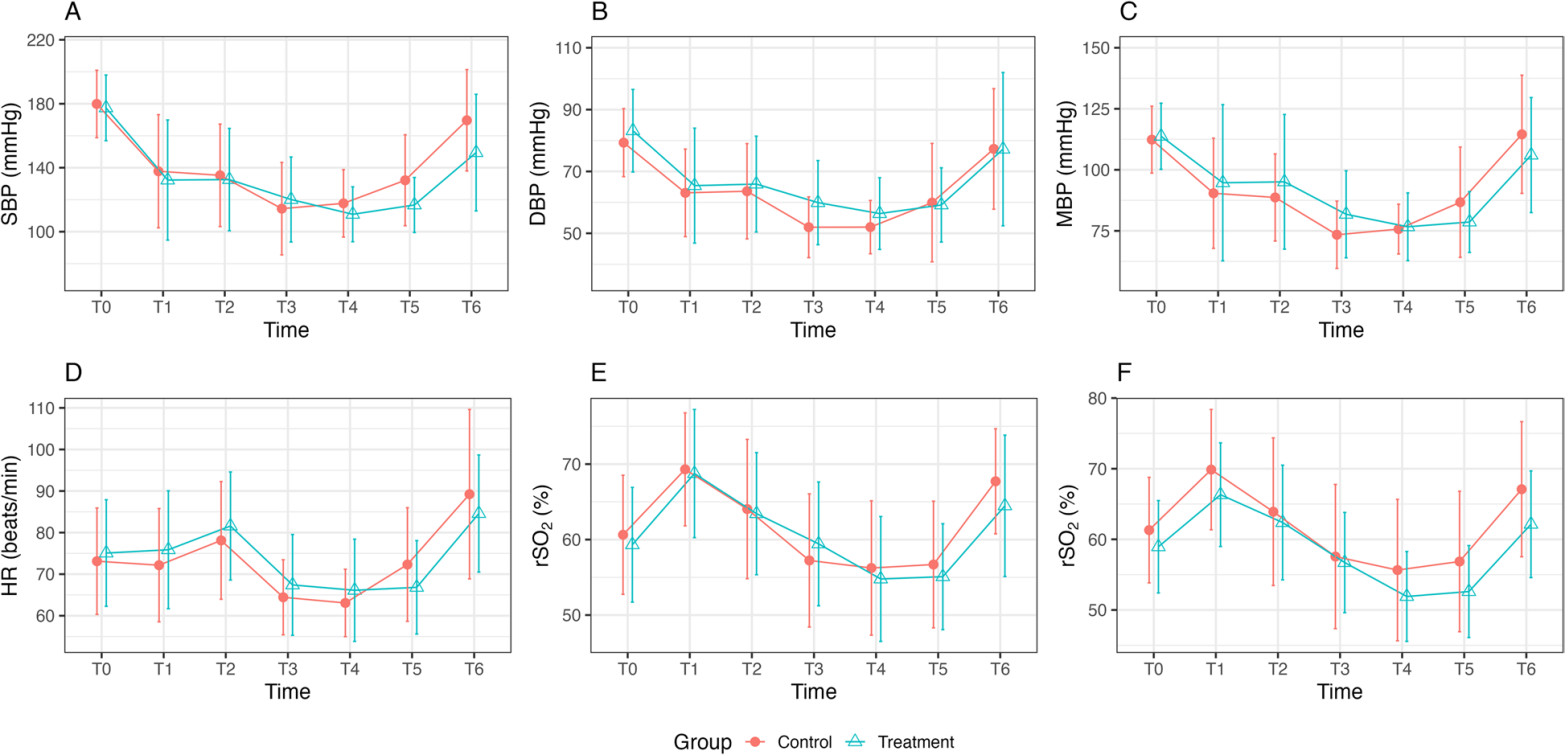
Changes in hemodynamics and cerebral oxygen saturation throughout the total shoulder surgery. Error bars represent mean ± SD. Serial changes of (**A**) systolic blood pressure (SBP), (**B**) diastolic blood pressure (DBP), (**C**) mean arterial pressure (MAP), (**D**) heart rate (HR), (**E**) left side of rSO_2_, and (**F**) right side of rSO_2_. T0, 1 min before induction; T1, 1 min before changing to the beach-chair position; T2, 1 min after changing to the beach-chair position; T3, 5 min after changing to the beach-chair position; T4, 30 min after changing to the beach-chair position; T5, 1 min before changing to the supine position; T6, 1 min after changing to the supine position

Figure 3 illustrates the hemodynamic measurements during surgery. SBP, DBP, and MAP demonstrated similar trends throughout the procedure with all blood pressure variables showing a significant drop after induction (all adj. *p* < 0.01). No significant changes were observed immediately before and after the transition to the beach chair position; however, a significant decrease was detected when comparing the values 1 min before and 5 min after the transition, except for DBP (adj. *p* = 0.02, 0.05, and 0.02, respectively).

HR did not change significantly after induction but showed a significant increase 1 min after changing to the beach chair position (adj. *p* = 0.04). HR then significantly decreased 5 min after the position change, below baseline (adj. *p* < 0.01).

rSO_2_ increased significantly on both sides after induction (both adj. *p* < 0.01). Subsequently, left rSO_2_ significantly decreased until 5 min after changing to the beach chair position, while right rSO_2_ significantly decreased until 30 min after the change. All hemodynamic variables showed a significant increase upon returning to supine position after surgery.

## Discussion

This study investigated the efficacy of leg elevation in preventing intraoperative hypotension and cerebral desaturation during total shoulder arthroplasty in the beach chair position. Our results indicated no significant differences in the incidence of intraoperative hypotension and cerebral desaturation, as well as in the frequency of these episodes per patient, between the control and treatment groups. However, patients with leg elevation required significantly less phenylephrine to manage hypotension, suggesting that leg elevation may reduce the severity and duration of intraoperative hypotension compared with the conventional leg position.

The association between hemodynamic instability and the beach chair position is well established. Transitioning from a supine to an upright or sitting position causes venous pooling in the lower extremities, reducing venous return to the heart. This gravitational shift can diminish stroke volume and cardiac output of up to 20% [3]. Several studies have investigated the effectiveness of different perioperative management strategies in mitigating hemodynamic instability during shoulder arthroplasty in the beach chair position [5–7]. Contrary to previous studies, our study focused on the efficacy of leg elevation as a standalone intervention to prevent hypotension and cerebral desaturation. Leg elevation is a simple, noninvasive, cost-effective, and safe technique that can temporarily increase the blood pressure. It facilitates the return of approximately 150–300 mL of venous blood from the lower extremities to the central circulation, resulting in a 10–15% increase in cardiac preload and cardiac output [11–15]. Based on these physiological effects, significant improvements in hemodynamic stability were expected in the treatment group compared with the control group. However, leg elevation did not reduce the incidence of hypotension, and no significant differences in the serial changes in hemodynamic variables were observed between the two groups throughout surgery.

A plausible explanation for our finding is that the hemodynamic benefits of leg elevation are both Limited in magnitude and short-lived. Previous studies on passive leg raising have demonstrated that the increase in stroke volume and the associated rise in blood pressure are transient, typically lasting only 7–10 min [11, 16]. In our study, these Limited effects were Likely further attenuated by the influence of general anesthesia. In non-anesthetized patients, postural changes trigger autonomic compensation, increasing systemic vascular resistance by 50–80% to preserve hemodynamic stability [17]. However, anesthetic agents impair these compensatory responses and further exacerbate hemodynamic instability through vasodilation and myocardial depression. Additionally, positive pressure ventilation further reduces venous return by increasing intrathoracic pressure, thereby compromising cardiac filling and output [18]. Taken together, these factors likely outweighed the modest and transient circulatory benefit provided by leg elevation, resulting in no significant differences between groups in the incidence of hypotension or cerebral desaturation, or in serial changes in blood pressure and cerebral oxygenation throughout the perioperative period.

Another contributing factor may be related to the definition of intraoperative hypotension and the duration of the observation period. In our study, hypotension was defined as a MAP < 60 mmHg or SBP < 80% of baseline sustained for at least one minute. This binary threshold may lack sensitivity to detect variations in the depth or cumulative burden of hypotensive events. Stratifying hypotension by severity or duration might have revealed more subtle group differences. Additionally, because the observation window extended from the initiation of the beach chair position to the end of surgery, any short-term benefit of leg elevation could have been diluted over time. A time-stratified analysis may have more clearly captured the temporal relationship between leg elevation and hypotensive episodes.

Despite the lack of a significant difference in the incidence of intraoperative hypotension or cerebral desaturation between groups, leg elevation appeared to exert a subtle yet favorable effect on hemodynamic stability. Because vasopressors were administered as needed to manage hypotensive episodes, a direct comparison of unmodified hemodynamic responses was not feasible. However, the control group required nearly twice the cumulative dose of phenylephrine compared to the leg elevation group, suggesting more frequent or prolonged hypotensive episodes in the absence of leg elevation. This finding implies that leg elevation may have helped attenuate the severity or duration of hemodynamic instability. While leg elevation may not serve as a primary method for preventing hypotension, its simplicity, safety, and low cost support its use as a practical adjunct in beach chair position surgeries, particularly benefiting patients with a higher risk of hemodynamic instability. Furthermore, previous studies have reported that phenylephrine, although effective in maintaining arterial pressure, may be associated with reductions in cerebral oxygenation and cardiac output in the beach chair position [6, 19]. A reduced requirement for phenylephrine could therefore offer potential clinical advantages. However, since no significant differences in cerebral oxygenation were observed between groups in our study, this reduction may not have translated into a measurable neuroprotective benefit. Further research is needed to clarify the clinical relevance of vasopressor dose reduction in this context.

In this study, a significantly higher incidence of hypotension was observed at 93.9% (46 of 49 patients), irrespective of leg elevation, compared to previous studies. This discrepancy is primarily attributed to differences in population selection. Various patient factors, such as old age, history of hypertension, and cardiovascular disease, significantly affect the incidence of hypotension due to increased sensitivity to anesthesia, decreased cardiac functional reserve, and autonomic nervous system dysfunction [20, 21]. Previous studies included subjects with average ages of 47 or 63 years and fewer underlying conditions [5, 10]. In contrast, the participants in our study had an average age of 71 years, with 83.7% of the patients having an ASA physical status of 2 or higher. Therefore, the study population generally had a higher susceptibility to intraoperative hypotension, leading to a higher observed incidence rate. Conducting the study with younger and healthier subjects might have minimized the confounding factors related to intraoperative hypotension and better evaluated the independent effect of leg elevation on hemodynamic stability. However, the objective was to reflect real-world circumstances by studying a representative population, as most patients undergoing total shoulder arthroplasty are elderly and have comorbidities [22–24].

Our study had several limitations. First, due to the nature of the intervention, blinding was not feasible, which may have introduced a potential bias. Second, essential hemodynamic parameters such as cardiac output, stroke volume, and transesophageal echocardiography-based preload measurements were not systemically assessed, limiting the comprehensive evaluation of cardiovascular responses. Third, patients with pre-existing cardiac or cerebrovascular diseases, who may be more susceptible to hemodynamic instability in the beach chair position, were excluded from the study. This exclusion may limit the generalizability of our findings to broader patient populations. Fourth, the absence of preoperative fluid status assessment might have influenced early intraoperative hypotension. Additionally, intraoperative fluid status was not evaluated using the stroke volume index or pleth variability index, which could have enabled more individualized fluid therapy and potentially influenced vasopressor usage. Finally, the study did not include postoperative follow-up, which is essential to evaluate the long-term effects and clinical relevance of intraoperative hypotension and cerebral desaturation. In particular, we did not assess postoperative clinical outcomes such as cognitive dysfunction or functional recovery, which could provide valuable insights into the potential consequences of impaired intraoperative cerebral perfusion.

In conclusion, leg elevation did not significantly reduce the incidence of intraoperative hypotension or cerebral desaturation; however, it contributed to hemodynamics stability by significantly reducing the use of phenylephrine.

## Supplementary Information

Supplementary Material 1

## Data Availability

The datasets used and/or analyzed during the current study are available from the corresponding author upon reasonable request.
